# A prospective multicenter phase I/II clinical trial to evaluate safety and efficacy of NOVOCART Disc plus autologous disc chondrocyte transplantation in the treatment of nucleotomized and degenerative lumbar disc to avoid secondary disease: study protocol for a randomized controlled trial

**DOI:** 10.1186/s13063-016-1239-y

**Published:** 2016-02-26

**Authors:** Anja Tschugg, Felix Michnacs, Martin Strowitzki, Hans Jörg Meisel, Claudius Thomé

**Affiliations:** Department of Neurosurgery, Innsbruck Medical University, Innsbruck, Austria; TETEC Tissue Engineering Technologies AG, Reutlingen, Germany; Neurocenter, Trauma Center Murnau, Murnau, Germany; Department of Neurosurgery, BG-Clinic Bergmannstrost, Halle, Germany

**Keywords:** Autologous disc chondrocyte transplantation, Degenerative disc disease, Lumbar back pain, Sequestrectomy

## Abstract

**Background:**

Intervertebral disc degeneration is emphasized as an important cause of low back pain. Current surgical treatment provides relief to the accompanying pain and disability but does not restore the biological function of the intervertebral disc. NOVOCART™ Disc plus, an autologous cell compound for autologous disc chondrocyte transplantation, was developed to reduce the degenerative sequelae after lumbar disc surgery or to prophylactically avoid degeneration in adjacent discs.

**Methods/design:**

This is a multicenter, randomized, controlled, clinical phase I/II combination study. A total of 120 adult patients are allocated in a ratio of 2:1:1. Sample size and power calculations were performed to detect the minimal clinically important difference of 10 units, with an expected standard deviation of 12 in the Oswestry Disability Index, which is the primary outcome parameter. Secondary outcome parameters include the visual analog scale and the EQ-5D questionnaire. Changes in physical and mental health are evaluated using the Short Form-12 (SF-12). Moreover, radiological and functional outcomes are evaluated. The major inclusion criterion is a single lumbar disc herniation that requires sequestrectomy. Transplantation is performed 90 days thereafter. Study data generation (study sites) and data storage, processing, and statistical analysis are clearly separated.

**Discussion:**

In this phase-I/II study, NDplus is being investigated for its clinical applicability, safety, and efficacy in the repair of herniated, nucleotomized discs, and of adjacent degenerated discs, if present. To date, autologous disc chondrocytes have not been transplanted into degenerative discs without previous disc herniation. As such, this is the first study to investigate a therapeutic as well as a prophylactic approach to treat degenerative discs of the lumbar spine.

**Trial registration:**

EudraCT No: 2010-023830-22, ID NCT01640457, 8 November 2010

## Background

The vast majority of low back pain (LBP) is self-limited or successfully treated conservatively, whereas a small proportion of patients develop chronic LBP intractable to nonsurgical treatments. These patients present an increasing medical and socioeconomic problem [1]. Intervertebral disc degeneration (IVDD) is emphasized as an important cause of LBP [2, 3]. With progressive degeneration, the effectiveness of the nutrition mechanism of the intervertebral disc decreases; in consequence, the nucleus pulposus cells lose their ability to produce proteoglycan and other important extracellular matrix proteins, which results in disc desiccation and progressive instability [4]. Most treatment approaches, for example, the aggressive removal of the pathological disc or spondylodesis, do not address options of restoring structural or biological deteriorations when IVDD is the underlying problem. However, cell retransplantation and restoration, as a procedure used in this trial, would offer a less invasive and biological alternative to lumbar fusion in an attempt to obviate the LBP associated with IVDD earlier in the degenerative cascade. Until now, an intervertebral disc may only be repaired biologically after a herniation through the transplantation of the patient’s disc chondrocytes [5–8].

Results of the application of ADCT showed that cellular revitalization of the intervertebral disc is possible [8], and the results of a prospective randomized and controlled multicenter trial for ADCT were promising [9]. For the survival and the adaptation of the cultivated cells to the harsh environment in the disc space, the availability of nutrients, the number of injected cells required to avoid nutritional problems due to cell overpopulation, and the pro-inflammatory environment play a crucial role. These problems should be overcome with a suitable biomaterial, which is used in this study [10].

By using an initially liquid biomaterial, which polymerizes after being injected, the cells adhere much better to the disc tissue. NOVOCART™ Disc plus (NDplus), an autologous cell compound for autologous disc chondrocyte transplantation (ADCT), was developed to provide rehydration and maintain the biological integrity of degenerative lumbar discs to prevent secondary diseases such as recurrent lumbar disc herniation, osteochondrosis, or segmental instability. The important properties of NDplus are anti-inflammatory, antiangiogenic and antiosteogenic. Moreover, NDplus is hydrophilic, supports cell morphology, and stabilizes the chrondrogenic phenotype.

In this phase-I/II study, NDplus is being investigated for its clinical applicability, safety, and efficacy in the repair of herniated, nucleotomized discs and of adjacent degenerated discs, if present.

The objective of this clinical study is to provide the basis for a confirmatory study design (endpoints and methodologies) (phase II) and to develop a safety profile for the surgical procedure as well as for NDplus (phase I). This study further aims at developing and validating known and new biologic markers for the quality and clinical efficacy of NDisc plus as requested in the context of identity, purity, and potency characteristics of the medicinal/investigational product.

## Methods/design

### Study design

The NOVOCART™ Disc study is a nonconfirmatory, prospective, multicenter, unmasked, randomized study with two phases aimed at gathering preliminary clinical information on NOVOCART™ Disc plus and NOVOCART™ Disc basic used in the treatment of a herniated disc. Phase I aimed to develop a safety profile of the investigational medicinal product NOVOCART™ Disc plus. The objectives of phase II are mainly to provide a basis for a confirmatory clinical trial design and proof of concept. Twelve trial sites are planned to participate in this combined phase-I/II study; three of them have already participated in phase I. In total, 120 patients meeting the inclusion criteria will be randomized, treated according to the randomization, and followed up for 5 years. The primary study endpoint of the phase II is the difference in the Oswestry Disability Index (ODI) between treatment groups 2 years after the intervention. This index has emerged as the most commonly recommended condition-specific outcome measure for spinal disorders. It is a self-administered questionnaire divided into 10 sections designed to assess the limitations of various activities of daily living [11, 12]. The Short Form-36 (SF-36) questionnaire and EuroQuality of Life-5 Dimension (EQ-5D) are applied to assess changes in health status and quality of life [13, 14]. Moreover, changes in leg and back pain are assessed by the Visual Analog Scale (VAS) [15]. Numerical measurements in the disc height, volumetry, and signal intensity of index and adjacent discs and categorical evaluations, such as degeneration scale and the presence or absence of stenosis, are determined by magnetic resonance imaging (MRI). Neurological and functional status and the quantity of current pain medication and time to return to work after surgery are documented. The serology of HIV, hepatitis and *Treponema palladium* are determined preoperatively. Laboratory values (IL-6, Leukotriene, and CRP) as safety vales are being evaluated only in phase I of the study. Adverse events and serious adverse events are documented. Histological investigation of the explanted tissue is performed. Primary and secondary outcome parameters are outlined in more detail in Table 1.

**Table 1:** Primary and secondary outcome parameters

Primary outcome parameter	Oswestry Disability Index (ODI), German and Austrian version, at 5 years’ follow-up to measure degenerative lumbar disc-related disability. [11, 12]
Secondary outcome parameter	Changes in health status and quality of life will be captured by the Short Form-36 (SF-36) version 2.0 questionnaire [14] and EuroQuality of Life 5-Dimension-3 L (EQ-5D-3 L) questionnaire [13]
	Pain relief, as defined by improvement on 100 mm visual analog scale for back and leg pain [15].
	Index level and adjacent disc on magnetic resonance imaging (MRI)
	• by evidence of disc degeneration (Pfirrmann Classification) or osteochondrosis (Modic Changes) [30–32]
	• by numerical measurements in disc high, volumetry, and signal intensity
	• by T2 relaxation
	• by the presence or absence of stenosis.
	Pain medication usage will be assessed by a four-level score (0 = none to 3 = high level of usage).
	Work status and time to return to work
	Neurological status will be evaluated using the Jenny Scale as a neurological test for the lower extremities, including motor and sensory deficits, reflexes, sciatic stretch ability and walking range.
	Functional status:
	• by posture and gait
	• by finger-ground distance
	• by Schober’s sign
	• by precision tenderness/pressure pain

### Trial organization, registration, and ethical aspects

Ethics approval was attained in Germany from the National Physician Board, “Landesärztekammer” Sachsen-Anhalt and in Austria from the committee of the Medical University Innsbruck. Furthermore, the clinical trial approval was obtained from the Paul-Ehrlich-Institute (Langen, Germany) and the Austrian Agency for Health and Food Safety (Vienna, Austria). The ethical bodies that approved the study in the various centers are listed in Table 2.

**Table 2:** Ethical bodies of the various centers

Study site	Ethical body
Innsbruck	Committee of the Medical University Innsbruck
Speising	Committee of the Medical University Vienna
Halle	National Physician Board Sachsen-Anhalt
Murnau	National Physician Board Munich
Münster	National Physician Board Westfahlen-Lippe
Kiel	Committee of the Medical University Kiel
Karlsruhe	National Physician Board Baden-Württemberg
Berlin	National Physician Board Berlin
Göttingen	Committee of theMedical University Göttingen
Idar-Oberstein	National Physician Board Mainz
Düsseldorf	Committee of the Medical University Düsseldorf

The study complies with the World Medical Association Declaration of Helsinki Ethical Principles for Medical Research Involving Human Subjects, Good Clinical Practice (GCP), national pharmaceutical acts in the participating countries (Austria and Germany), and European guidelines for the conduct of clinical trials with medicinal products for human use. The trial is initiated and sponsored by TETEC Ag (B|Braun Aesculap AG shared company, Reutlingen, Germany), which is responsible for management and registration (EudraCT No: 2010-023830-22, ID NCT01640457). CenTrial GmbH (Tuebingen, Germany) is responsible for clinical trial submission, independent clinical monitoring, and pharmacovigilance. Mediri GmbH (Heidelberg, Germany), as a core imaging lab, is responsible for the development of the MRI protocols, data storage and analyses. Laboratory values are investigated centrally by Synlab Services GmbH (Synlab MVZ, Leinfelden-Echterdingen, Germany). Accovion GmbH (Eschborn, Germany) is responsible for data management, biometrics, and medial writing. Quality assurance is being done in cooperation with participating clinical research organizations (CRO).

### Study population

The N-Disc trial aims to include patients who qualify for lumbar sequestrectomy because of a lumbar disc herniation. The target population consists of patients with symptomatic lumbar disc herniation who failed adequate conservative or interventional treatment approaches in accordance with the guidelines of DGNC and DGOOC. Additionally, an MRI-determined lesion at treatment level needs to correlate with the primary symptoms. To minimize risk factors, patients with significant comorbidities have been excluded from the study. Written informed consent has been obtained from each patient. Inclusion and exclusion criteria are outlined in more detail in Table 3.

**Table 3:** Inclusion and exclusion criteria

Initial inclusion criteria	A disc herniation with back and/or leg pain (radicular pain)
	Indication for sequestrectomy according to the guidelines of German Society of Neurosurgery and the German Society of Orthopedics and Orthopedic Surgery
	Age between 18 and 60 years
	Physically and mentally able to participate in the study and able to understand the study, its goals, and the possible risk factors involved
	Willing and able to participate in the follow-up visit plan at the study site and able to understand and to complete a study-relevant questionnaire in German language
	Sufficiently informed about this trial orally and in writing. She/he had enough time for consideration, is willing to participate in the study, and gives her/his written informed consent
	No participation in a clinical study 90 days prior to study inclusion. She/he agrees to refrain from participating in another clinical study during the NOVOCART™ Disc Study and for another 90 days after study termination
Initial exclusion criteria	Previous surgery at the lumbar level(s) and has been treated with NOVOCART™ Disc plus or NOVOCART™ Disc basic
	Recurrent disc herniation treated with nucleotomy/sequestrectomy of the relevant disc
	Degenerative muscular or neurological conditions that would interfere with evaluation of outcome measures, including but not limited to Parkinson’s disease, amyotropic lateral sclerosis, multiple sclerosis, muscular dystrophy, and myelopathic diseases of different causes
	Body Mass Index?>?35 kg/m2
	Current or recent history of illicit drug, nicotine (more than 20 cigarettes per day) or alcohol abuse or dependence
	CRP?>?10 mg/dl
	Pregnant, breastfeeding or planning to become pregnant. Female patients must be either at least 2 years postmenopausal or using one of the following means of birth control during the treatment phase, that is, for transplantation
	• surgical sterility
	• double barrier methods, for example, condom or diaphragm in combination with spermicide
	• intrauterine contraceptive device
	• bilateral vasectomy of sexual partner at least 90 days prior to enrollment in combination with barrier methods (for example, condom or diaphragm)
	• birth control pill
	History of known allergies or a suspicion of allergies to any of the NOVOCART™ Disc plus or basic product components, including hyaluronan, polyethylene glycol or albumin
	Immune defects or the affinity for infections of known or unknown causes
	Active systematic or local microbial infection, eczematization, or inflammable skin alterations at the site of surgery
	Unable to undergo magnetic resonance imaging
	History or suspicion of a disease with chronically inflammable character, as rheumatoid arthritis, gout, pseudo-gout, metabolic bone diseases, Crohn’s disease, ulcerative colitis, lupus erythematosus, or other autoimmune disorders
	Known osteoporosis
	Primary hyperparathyroidism or hyperthyroidism, chronic renal failure, or previous fragility fractures
	Systematic connective tissue or collagen disease
	Hereditary ocular degeneration with unclear diagnosis, retinopathies based on connective tissue-defined causes, macular corneal dystrophy
	Immune suppression
	History of blood coagulation disease of different genesis, including known hemorrhagic diathesis of unknown cause
	Chemo or radiotherapy within the past 5 years or had any cancer other than nonmelanoma skin cancer treated with curative intent within the past 5 years
	Known diabetes, drug treated
	Ulterior concomitant diseases or functional impairments of specific organs, which exclude study participation by the assessment of the investigator
Radiological inclusion criteria	Single level lumbar disc herniation
	More than 50 % remaining disc height in the herniated disc in comparison to unaffected discs in the lumbar spine. If all discs show degenerative signs, disc height must be at least 5 mm
	No obvious signs of osteophytes and no endplate sclerosis in the lumbar segment to be treated with NOVOCART™ Disc plus or NOVOCART™ Disc basic
	Patients without adjacent degenerative discs (HD): adjacent proximal disc has no degenerative signs according to Pfirrmann Score stage 3 to 5
	Patients with adjacent degenerative discs (AAD): additional degenerative signs in the proximal adjacent lumbar level according to Pfirrmann 3 to 4 but no more than 25 % disc height reduction
Radiological exclusion criteria	Degenerative changes in the lumbar spine as determined by Modic Changes 2 to 3
	One or more dysplastic vertebral bodies within the lumbar spine
	Sacralized lumbar vertebra LWK 5 at the level to be treated with NOVOCART™ Disc plus or NOVOCART™ Disc basic
	Previous or acute spondylodiscitis
	Segmental instability (spondylolisthesis?>?5 mm) or translation?>?3 mm
	Isthmic spondylolisthesis, ankylosing spondylitis, or spondylolysis
	Lumbar scoliosis (>11° deformation)
	Previous trauma, discography or any other surgical intervention at the lumbar spine
	Previous compression or burst fracture at the level(s) to be treated with NOVOCART™ Disc plus or NOVOCART™ Disc basic
	Central spinal canal stenosis with evidence of a narrowing of?<?8 mm (by sagittal MRI)
	Spinal tumor
	Metabolic bone disease
	Facet ankylosis or severe facet degeneration
	Lumbar kyphosis
Intra-surgery exclusion criteria	Extensive damage of annulus, which subsequently poses a significantly greater risk of recurrence
Exclusion criteria after sequestrectomy	HIV, *Treponema pallidum*, active hepatitis B or C infection
Exclusion criteria prior transplantation	Recurrent disc herniation after surgery and prior transplantation

### Timetable

**Fig. 1:**
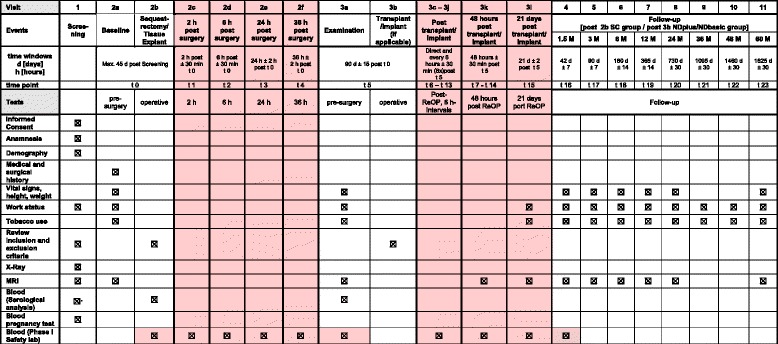
Visit plan. In phase I, close monitoring of the safety parameters and an additional assessment of the magnetic resonance imaging (MRI) will occur

The visit plan is outlined in Fig. 1. In phase I, close monitoring of the safety parameters and additional assessment of the MRI will occur.

### Investigational medicinal product

NOVOCART™ Disc plus (NDisc), an autologous cell compound for ADCT, was developed to provide rehydration and maintain the biological integrity of degenerative lumbar discs to prevent secondary diseases such as recurrent lumbar disc herniation, osteochondrosis, or segmental instability. NDisc plus is an injectable, in situ-polymerizing, modified albumin, hyaluronic acid gel with ex vivo-expanded autologous disc derived chondrocyte cells as the active ingredient. NDisc plus is composed of two components: Component A is a suspension with 3.6 to 4.4 million autologous intervertebral disc cells contained in a solution of modified human albumin, human serum, culture media components, chondroitin sulfate, and hyaluronic acid. The total volume is 4 ml suspension placed in a 6 ml glass vial. Component B is a bis thio-polyethylene glycol (BTPEG) solution; the total volume is 1 ml in a 2 ml glass vial. Components A and B polymerize in situ using a dedicated application system (special dual-chamber syringe) to form the desired hydrogel. The preparation is for single use during the ADCT. The dosage is individual, and the volume of injection is, depending on the capacity of the treated disc, between 0.5 and 2 ml cell suspension volume. NDisc basic is used as the placebo in the NDisc study and is the cell-free hydrogel carrier material with an identical composition but without growth factors.

### Sequestrectomy

Surgery is performed by two trial-designated surgeons with the assistance of an operating microscope while the patient is under general endotracheal anesthesia and in a prone position. Depending on the location of the disc herniation, the spinal canal harboring the sequestrated disc material is exposed by either a minimal interlaminar fenestration or a translaminar approach [16]. In case of a lateral lumbar disc herniation, a lateral extraforaminal approach is performed [17]. Immediately after extraction, the disc tissue is transferred into a sterile transport vial and provided to TETEC AG for the GMP compliant manufacturing of the investigational medicinal product NDisc.

### Adjacent disc disease

The presents or absence of an adjacent degenerative disc is thought to be a potential factor affecting the prophylactic treatment capacity of NDisc plus. Analyses of primary and secondary efficacy variables will be performed separately for these two patient subgroups in addition to the overall analysis.

### Transplantation

**Fig. 2:**
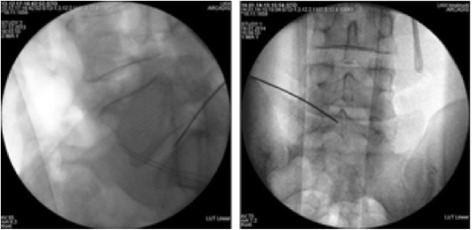
Transplantation. An injection needle is placed in the center of the disc space contra-laterally to the side of the disc surgery. In case of an adjacent disc disease, positioning of the needle takes place simultaneously to minimize radiation exposure

Transplantation is performed 90 days after sequestrectomy. N Disc plus (NDplus) or N Disc basic (NDbasic) is applied via an injection with a dual-needle technique directly at the intended site of action. NDplus or NDbasic is to be transplanted in a comfortable lateral or abdominal position. After the treatment level is localized and local anesthesia is performed, the puncture of the intervertebral disc concerned takes place contralaterally to the disc surgery. An injection needle is placed in the center of the disc space under image guidance, and the medicinal product is injected. In case of an ADD (adjacent disc degeneration), positioning of the needles and the mandrin takes place simultaneously to minimize radiation exposure (Fig. 2).

### Randomization

**Fig. 3:**
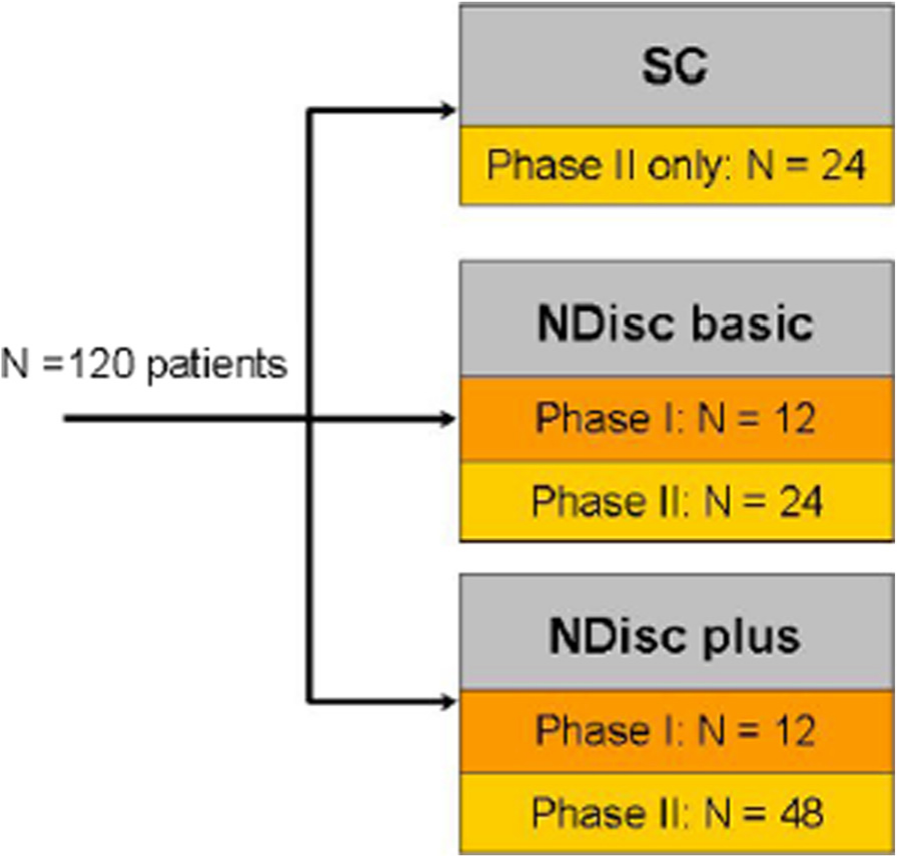
Randomization. Randomization is performed separately for phase I and phase II of the study

Randomization is performed intraoperatively by envelope and separately for phase I and phase II of the study. In phase I, 24 patients are randomly assigned to NDplus and NDbasic in a 1:1 allocation ratio. In phase II, 96 patients are randomly assigned to NDplus, NDbasic or follow the standard of care treatment (surgery only, SC). The allocation ratio in phase II is 2:1:1. Altogether, 60 patients will be assigned to NDplus, 36 to NDbasic, and 24 to SC (Fig. 3). Two randomization schedules are generated by CROs using Rando™ (CRO’s proprietary randomization software). The randomization schedule is kept at the CRO by the randomization code administrator, who is independent from the study team. A copy of the randomization schedule is provided to TETEC AG.

### Sample size and power calculation

The sample size calculation is based on the primary endpoint of the study, which is the group difference in the ODI. A change of 10 units is considered as a clinically meaningful difference. Moreover, a standard deviation of 12 is assumed, based on results of a previous trial (Hägg, Fritzell, Nordwall, 2003). A sample size calculation was performed based on a two-group t-test (nQuery Advisor™ Software, version 7.0). The width of the respective confidence intervals were calculated using a large sample approximation, with 60 patients to be enrolled in the NDplus group, 36 patients in the NDbasic group, and 24 patients in the SC arm (120 patients in total). A detailed power calculation is shown in Table 4.

**Table 4:** Power calculation

Treatment group mean / Treatment effect (contrast)	Width of two-sided 95 % confidence interval from point estimate to limit (units in standardized ODI)		Power of pairwise t-test to detect a difference in change from baseline of 10 units in ODI
	ODI sum score (SD = 12 points)	Change in ODI (SD = 14.7 points = 12*sqrt (2(1-r)) when *r* = 0.25	Change in ODI (SD = 14.7 points = 12*sqrt (2(1-r)) when *r* = 0.25
SC (*n* = 24)	4.80	5.88	–
NDbasic (*n* = 36)	3.92	4.80	–
NDplus (*n* = 60)	3.04	3.72	–
NDbasic (*n* = 36) versus SC (*n* = 24)	6.20	7.59	71 %
NDplus (*n* = 60) versus SC (*n* = 24)	5.68	6.96	79 %
NDplus (*n* = 60) versus NDbasic (*n* = 36)	4.96	6.07	89 %

### Statistical analysis

#### Efficacy variables

The analysis of efficacy variables will focus on estimation of treatment effects and differences between treatment groups. For this purpose, it is assumed that effects of treatment with SC, NDbasic, and NDplus are additive so that treatment effects can be assessed by location shifts (like mean change or median change) between treatment groups. Differences between NDbasic and SC are the total effect of NDbasic, the transplantation/implantation procedure to inject the NDbasic, and the time interval between tissue harvest and transplantation/implantation. Observed differences between NDplus and SC are the total effect of NDplus, the chondrocytes, the transplantation procedure to inject the NDplus, and the time interval between tissue harvest and cell transplantation. Consequently, observed differences between NDplus and NDbasic are assumed the sole effect of chondrocytes. All efficacy data will be presented for patients in the Full Analysis Set (FAS). Primary and secondary efficacy variables and their changes from baseline will be summarized by treatment group. An analysis of variance with factors treatment and the presence or absence of a degenerative adjacent disc (ADD/HD) will be used to evaluate continuous and quasi-continuous, approximately normal data. Confidence intervals will be based on estimated means (least square means) and the corresponding t-statistic. Additional covariates, such as baseline measurements, will be included in the model as appropriate. VAS readings will be summarized by treatment group and visit over the course of the study. MRI parameters and variables to assess functional neurological status will be summarized descriptively. Survival analysis will be used to evaluate time to return to work after surgery. The Kaplan-Meier product-limit estimator will be used to estimate the probability to return to work over time by treatment group for all patients in the FAS. Imputation of missing data has not been planned.

#### Safety variables

Adverse events (AEs) will be coded according to the latest Medical Dictionary for Regulatory Activities (MedDRA™) version available at the day of database closure. The analysis of adverse events will focus on treatment-emergent adverse events (TEAE). The frequency and percentage of TEAEs and possibly related TEAEs (that is, any possibly, probably, or definitely related TEAEs) will be summarized according to primary system organ class and preferred term and tabulated by treatment group. All laboratory values will be classified as normal or abnormal according to the laboratories’ normal ranges. Morphological parameters like the Modic Score, the detection of an extra discal fluid collection and re-herniation of the treated disc will be monitored by a MRI protocol. An independent data safety monitoring board (DSMB) has reviewed the phase I data, and no concerns have been raised. An ongoing evaluation by the DSMB will be undertaken during phase II. If relevant concerns should be detectable like increased reherniation rates, a discontinuation of the trial must be considered.

#### Interim and final analysis

Data collected in the study may be analyzed and reviewed continuously. Early findings may be used to stop the study in case of safety concerns or lack of efficacy or futility with regard to a positive outcome in the NDplus group. To ensure the efficient use of clinical data, interim analyses are being performed (see Table 5).

**Table 5:** Interim and final analyses

1st interim analyses	Early evaluation for safety and feasibility, when all 24 patients of phase I are randomized and tissue explants, implant procedures, and 6-week follow-up visits are completed.
2nd interim analyses	Final evaluation for surgical feasibility of the procedures, when all enrolled patients have completed the sequestrectomy and transplantation.
3rd interim analyses	Early evaluation for efficacy with patients who have completed the 12-month follow-up visit.
4th interim analyses	Primary evaluation for efficacy with all patients who have completed the 24-month follow-up visit.
Final analyses	When all patients have completed the scheduled 60-month follow-up visit.

## Discussion

Next to the high prevalence of lumbar back pain caused by degenerative disc disease and the increasing number of spinal interventions [1], the major advantage of disc repair procedures is the ability to address the source of low back pain with a minimally invasive technique. Autologous chondrocyte cell transplantation has been shown to reduce intervertebral disc degeneration in animal models, as well as to be safely applicable in humans [9, 18, 19].

The avascularity of the lumbar disc is a promising precondition for autologous chondrocyte cell transplantation. Limited blood flow inhibits cell migration and provides an immunologically privileged environment [20]. Controversially, transplanted cells must survive in a limited blood supply and in a mechanically stressful environment that can cause a loss of native chondrocyte cells and leads to a decreased extracellular matrix production [4]. Therefore, the choice of biomaterial is important.

By using an initially liquid biomaterial, which polymerizes after being injected into the intervertebral disc, the cells adhere much better to the disc tissue. In an animal experiment, a significant increase in the survival rate of cells that were injected into the intervertebral disc was achieved with a polymerizing biomaterial as opposed to the application in a watery solution [10].

The use of hyaluronic acid and chondroitin sulfate in a defined concentration and composition stabilizes the phenotype of the expanded cells and allows an environmental conditioning, a more balanced nutrient distribution, and ultimately a higher cell survival rate after transplantation, by anti-inflammatory and osmotic effects [21–23]. Furthermore, the polymerizing hydrogel has anti-osteogenic and anti-angiogenic effects [24].

The hydrodynamic properties of hyaluronic acid have been known for many years [25]. Glycosaminoglycan plays a major role in the extracellular matrix of the healthy human intervertebral disc [26]. In an intervertebral disc with degenerative changes, however, the proteoglycans, the level of hyaluronic acid, and subsequently, the fluid content are already substantially reduced [27, 28]. Besides a high bio- and cell compatibility, a polymerizable biomaterial must also possess other important properties in order to be suitable for the biological disc reconstruction. It must ensure that the biomaterial stays in the disc after its in vivo polymerization and during the remobilization of the patient, that is, during increased physical stress [29].

The goal of ADCT is to reduce the degenerative sequelae after lumbar disc surgery or to prophylactically avoid degeneration in adjacent discs. Injection of material into the disc, however, theoretically carries several risks, such as extrusion of the injected material, increased intradiscal pressure, potentially provoking disc herniations, and local or systemic inflammatory reactions.

### Trial status

Phase I of the trial started in October 2012 with one site in Austria (the Department of Neurosurgery, Medical University Innsbruck), followed by two sites in Germany (the Department of Neurosurgery, BG Clinic Halle-Bergmannstrost (February 2013) and Department of Neurosurgery, BG-Clinic Murnau (March 2013)). Phase I revealed no relevant safety issues as reviewed by an independent DSMB. Thus, phase-II was planned with the following nine sites in Germany and Austria contributing: the Department of Neurosurgery, Medical University Düsseldorf; Department of Orthopedics-II, St. Franziskus Hospital Muenster; Department of Neurosurgery, Medical University Charité Berlin; Center of Spine Surgery, DRK Clinic Berlin Westend; Department of Neurosurgery, City Clinic Karlsruhe; Department of Neurosurgery, SHG Clinic Idar-Oberstein; Department of Trauma Surgery, Medical University Göttingen; Department of Neurosurgery, Medical University Schlewig-Holstein (Kiel); and Department of Spine Surgery, Orthopedic Hospital Speising.

## Abbreviations

ADCT: autologous disc chondrocyte transplantation
ADD: adjacent disc degeneration
BTPEG: bis thio-polyethylene glycol
CRO: clinical research organizations
CRP: C-reactive protein
EQ-5D: EuroQuality of Life-5 Dimension
FAS: Full Analysis Set
GCP: Good Clinical Practice
IL: Interleukine
IVDD: intervertebral disc degeneration
LBP: low back pain
MedDRA™: Medical Dictionary for Regulatory Activities
MRI: magnetic resonance imaging
NDplus: NOVOCART™ Disc plus
SF-12: Short Form-12
ODI: Oswestry Disability Index
TEAE: treatment emergent adverse events
VAS: Visual Analog Scale
